# Modeling of Interfacial Tension and Inclusion Motion Behavior in Steelmaking Continuous Casting Mold

**DOI:** 10.3390/ma16030968

**Published:** 2023-01-20

**Authors:** Md Irfanul Haque Siddiqui, Latif Arifudin, Ibrahim Abdullah Alnaser, Masood Ashraf Ali, Khalid Alluhydan

**Affiliations:** 1 Department of Mechanical Engineering, King Saud University, Riyadh 11451, Saudi Arabia; 2 Center of Excellence for Research in Engineering Material (CEREM), King Saud University, Riyadh 11451, Saudi Arabia; 3 Department of Industrial Engineering, College of Engineering, Prince Sattam Bin Abdulaziz University, Al-Kharj 16273, Saudi Arabia

**Keywords:** interfacial tension, clean steelmaking, modeling and simulation, inclusion, sulfur, alumina, SPFH590

## Abstract

The current work is an expansion of our previous numerical model in which we investigated the motion behavior of mold inclusions in the presence of interfacial tension effects. In this paper, we used computational fluid dynamic simulations to examine the influence of interfacial tension on inclusion motion behavior near to the solid–liquid interface (solidifying shell). We have used a multiphase model in which molten steel (SPFH590), sulfur, and alumina inclusions have been considered as different phases. In addition, we assume minimal to negligible velocity at the solid–liquid interface, and we restrict the numerical simulation to only include critical phenomena like heat transport and interfacial tension distribution in two-dimensional space. The two-phase simulation of molten steel mixed with sulfur and alumina was modeled on volume of fluid (VOF) method. Furthermore, the concentration of the surfactant (sulfur) in molten steel was defined using a species model. The surfactant concentration and temperature affect the Marangoni forces, and subsequently affects the interfacial tension applied on inclusion particles. It was found that the alteration in interfacial tension causes the inclusion particles to be pushed and swallowed near the solidifying boundaries. In addition, we have compared the computational results of interfacial tension, and it was found to be in good agreement with experimental correlations.

## 1. Introduction

**Figure 1:**
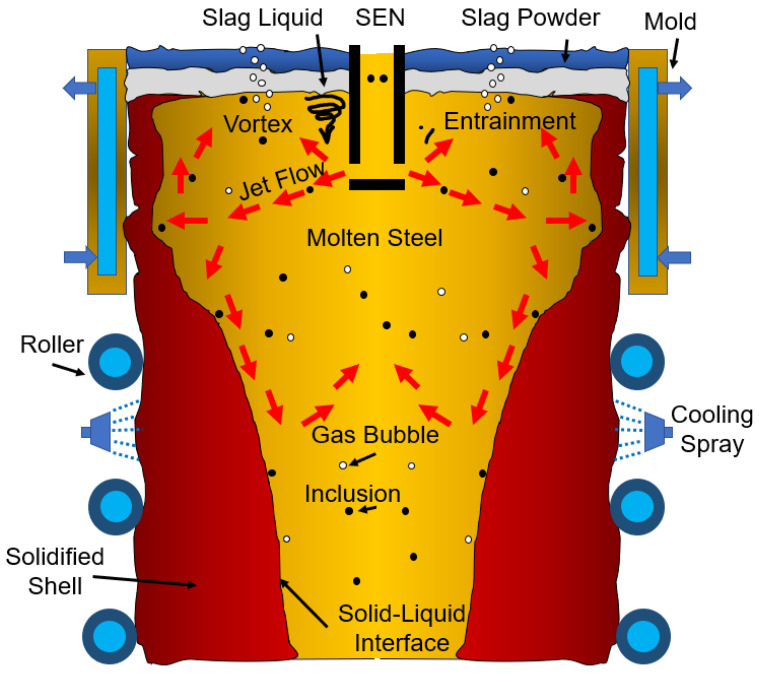
Schematic diagram of the continuous casting mold.

The steelmaking process is a complex process that involves the extraction of iron ore and carbon from the ground, which are then combined in a blast furnace. Clean steelmaking is an important part of the manufacturing process. It allows for sustainable, environmentally friendly production of steel, and minimizes harmful byproducts from the manufacturing process. The steelmaking process is one of the most energy-intensive in the world, but it can have a positive impact on the environment by making clean steel for sustainable future applications. Despite its lengthy social and technological history, the steel industry is still one of the most rapidly growing sectors that affects every part of our lives. Much work has gone into improving the surface and inner quality of continuously cast products to provide a better premium cast metal yield [1,2,3,4,5]. To keep up with the expanding capacity of producing raw steel, continuous casting productivity was also greatly increased. The production of carbon and specialty steels by continuous casting currently exceeds 95%. Concerns about cost, energy use, and the environment in the production process are becoming more significant since steel’s mechanical properties and chemical composition are constantly changing. Steel’s durability, corrosion resistance, ductility, and strength have all improved over time to meet demand. Since steel was first made commercially available at the turn of the 20th century, it has been extensively used for a variety of purposes because of its superior qualities [6]. It is possible to create molten steel from waste steel or iron ore, which is an alloy largely composed of the elements carbon and iron (i.e., scrap). In light of competitiveness, consumer demands, sustainability, greenhouse gas emissions, and other factors, the need for clean steel is a crucial issue for steelmakers. Non-metallic impurities have a big impact on steel goods’ quality, according to several reports. The mold remains the last stage [7] in the continuous casting process where the proper implication of processes can lead to significant inclusion removal. It is critical to comprehend the inclusion motion behavior in the mold as depicted in Figure 1 to effectively regulate non-metallic inclusions during continuous casting operations. The final steel products’ characteristics would be compromised by inclusion caught by the solidifying front during continuous casting [6,8,9].

### 1.1. Impurities in Steel

In steel, there are two different types of nonmetallic inclusions, each of which forms differently. One type is indigenous oxide inclusions, which appear when the steel melt is deoxidized. While refining and ladle degassing remove the majority of these oxides from the melt, certain non-metallic oxide inclusions are not removed. Exogenous inclusions develop when de-oxidized steel melt is re-oxidized by air or when entrained slag is added to the melt during the transfer of the melt from the ladle to the mold. Exogenous inclusions tend to be bigger than native inclusions and are therefore more dangerous. During the casting, rolling, and heat-treating procedures, inclusions pose issues and run the risk of the steel breaking [10,11,12,13]. The size and nature of nonmetallic inclusions that decrease steel properties are not constant, but rather fluctuate according to the application [14,15,16,17,18,19,20]. Changing the chemical makeup of large inclusions to reduce their melting temperature and make them malleable during hot rolling is another way for mitigating the detrimental consequences of large inclusions. The steel matrix elongates these big inclusions into thin solidified strips as it rolls. Because thin solidified strips are fragile at cold rolling temperatures, they shatter into little pieces when hot-rolled steel is cold-rolled. An undesired big inclusion may be divided into numerous smaller, innocuous inclusions when the space between the shattered pieces is prolonged by changing the deformability. Impurities in the melt that dissolve and precipitate during solidification must be minimized as well. Aluminum deoxidation typically removes oxygen contaminants during the secondary steelmaking process. However, the deoxidation process results in the formation of alumina inclusions. To guarantee that steel components and structures work as intended, alumina inclusions must be controlled [21,22,23,24]. During continuous casting and hot rolling, improper removal of alumina inclusions during the steel-making process can cause micro-surface cracks [14,25,26]. In general, liquid steel does not moisten solid nonmetallic impurities very effectively. These inclusions that are dispersed throughout the liquid steel might occasionally become trapped in the mold’s solidification interface and turn into flaws in the finished steel products. Therefore, it is important to thoroughly explore how such inclusions behave at the interface.

### 1.2. Effect of Interfacial Tension

**Figure 2:**
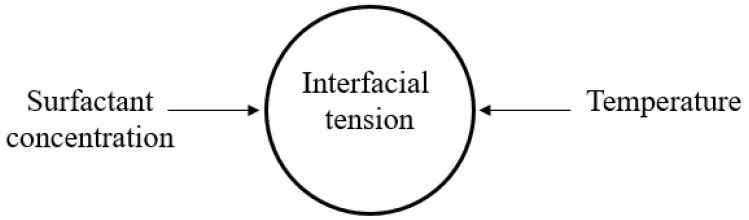
Variables affecting interfacial tension.

**Figure 3:**
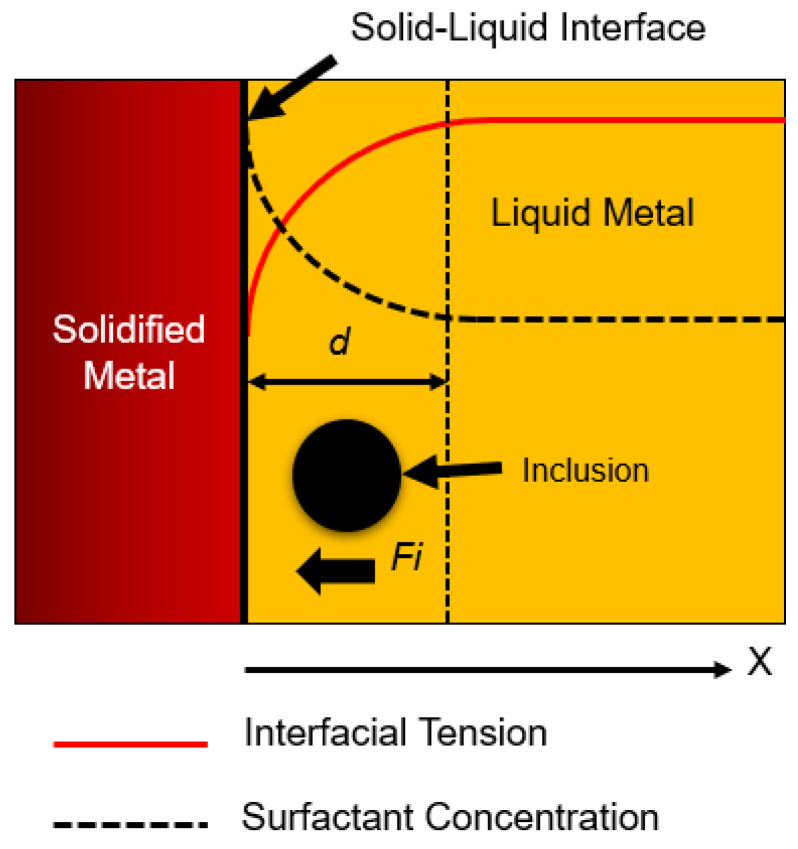
Interfacial tension and surfactant concentration on inclusion near the solid–liquid interface.

The Marangoni convection in the molten metal and the subsequent solidification in the mold are significantly influenced by the concentration of minor elements in steels. The major metals, such as chromium, manganese, nickel, phosphorus, silicon, and sulfur have a strong effect on the interfacial tensions. An increase in these minor elements will result in changes in the interfacial gradient. The presence of these elements influences the convection process, and affects the shape of the solidification front and its speed. The Marangoni force often referred to as the pushing and engulfment phenomenon [27,28,29,30,31] and subsequent interfacial tension influence the distribution of inclusion particles retained in the solidified slab of the continuous casting mold. The Marangoni force is a result of the solid–liquid interface between two substances. The more concentrated the solution, the higher the Marangoni force. Figure 2 provides an illustration of the factors influencing interfacial tension. Interfacial characteristics have an impact on how inclusion particles are pushed and engulfed [32,33,34]. When there is an interfacial tension gradient surrounding a gas bubble or a tiny liquid inclusion, the particles shift from one point of greater interfacial tension to another position of lower interfacial tension. The Marangoni force’s impact on inclusions at the border between solids and liquids is depicted in Figure 3. In general, surface tension decreases as temperature rises, just as it does with pure metals. Whenever a metal possesses the surface-active element, however, this is not always the case. The Marangoni convection in the molten metal and the subsequent solidification in the mold are significantly influenced by the concentration of minor elements in steels [35,36,37,38,39,40,41,42,43,44,45,46,47].

In water model studies, several researchers have studied this phenomenon in detail using bubbles. According to Mukai et al. [32,33,34] the gradient of interfacial tension present at the boundary layer causes the pushing and engulfment of tiny bubbles to happen in the solid–liquid interface. Shibata et al. [10] also investigated the motion behavior of inclusions and bubbles at the solid–liquid interface of solidifying metal surfaces. Researchers have emphasized that the Marangoni effect, which is brought about by thermal gradients or concentration gradients of surfactant components, would have an impact on the entire process of microgravity studies [30,35]. Even with low oxygen and sulfur concentrations, Marangoni flow was extremely strong in molten steel, according to Yin and Emi [30]. Nakamoto et al. [48] investigated the impact of inclusion wettability and interfacial tension on the immersion nozzle. By analyzing the interfacial tension and wettability phenomenon between the alumina sample and molten iron, they developed an experimental correlation for the interfacial tension. A comprehensive experimental study was conducted by J. Jeong et al. in 2020 [26] to determine the relationship for the interfacial tension between an alumina inclusion and liquid steel. They investigated the impact of temperature, sulfur, and surfactant concentration on interfacial tension. Further, Furukawa, Saito, and Nakashima [39] investigated the wettability of three different non-metallic inclusions namely, Al_2_O_3_, MgO, and MgO·Al_2_O_3_ substrates, concerning molten Fe and molten high-alloy steel SUS304 (Fe–18%Cr–9%Ni). They mentioned that the change in surface tension was caused by dissolved surfactant oxygen on the surface. In addition to this, Shen et al. [49] investigated the wettability of an iron and aluminum alloy and sintered MgO substrate. They mentioned that the contact angle between the sintered MgO substrate and the liquid iron was marginally influenced by aluminum concentrations of 18 ppm and 370 ppm in the liquid iron.

The present work is an extension of our previous numerical model [50] where we studied the inclusions’ motion behavior in the mold under the influence of interfacial tension. In our previous work [50], we investigated a full-scale model of the mold to simulate the melt flow and inclusion flow characteristics depending on some fundamental parameters. In the present research work, we investigated the effect of interfacial tension on inclusion motion near the solid–liquid interface (solidifying shell) by simulation. The continuous casting mold and molten automobile steel (SPFH590) have been the main subjects of the research. The alloying components of steel that will be modeled for this study are listed in Table 1. This work assumes minimal to the negligible velocity at the solid–liquid interface and restricts the numerical simulation to only include critical phenomena like heat transport and interfacial tension distribution in a two-dimensional space. Only a few millimeters of domain size have been studied in terms of inclusion motion at the solid–liquid interface. A further species model was utilized to establish surfactant concentration, and the volume of fluid (VOF) approach was chosen for two-phase simulations. Therefore, at various sulfur concentrations and temperatures, inclusion motion may be properly investigated. Details of the thermo-physical characteristics that were used for numerical modeling are included in Table 2 and Table 3.

## 2. Mathematical Modeling

To simulate the flow of molten steel and the motion of alumina inclusions in the continuous casting mold, a computational fluid dynamic (CFD)-based numerical model was developed using Ansys Fluent. The liquid steel flow, heat transmission, solidification of molten steel, and inclusion motion have all been taken into account by the simulation model. As a result, the simulation was done using a two-dimensional model. The study’s many characteristics, including surfactant content and molten metal temperature, were all taken into consideration.

The study of J. Jeong et al. [25] and Siddiqui et al. [53] has been taken into account for the experimental results on the interfacial tension of alumina inclusions as well as other thermal and physical properties.

### 2.1. The Governing Equation

To simulate melt flow and anticipate inclusion motion in continuous casting molds, a numerical model was devised. In this study, we employed a simple geometry to analyze the mobility of an alumina inclusion close to a solid barrier. To examine the impact of various interfacial phenomena in this scenario, a two-dimensional, laminar, multiphase model was built alongside several other models. The mathematical model for mass and momentum for the current simulation model, which was developed using a mathematical model based on CFD, are as follows:

(1) $$\frac{\partial \rho }{\partial t}+\nabla \cdot \left(\rho \vec{v}\right)=S_{m}$$

(2) $$\frac{\partial }{\partial t}\left(\rho \vec{v}\right)+\nabla \cdot \left(\rho v\vec{v}\right)=-\nabla p+\nabla \cdot \left(\overset{=}{\tau }\right)+\rho \vec{g}+\vec{F}$$

where,

$\overset{=}{\tau }$: stress tensor;
*p*: static pressure;
$\vec{F}\;$: external body force;
$\rho \vec{g}$: gravitational body force;
*S_m_*: mass source term.

The velocity field, pressure, and temperature of the liquid in the specified domain are determined using the solutions of the aforementioned equations. The stress tensor $\overset{=}{\tau }$ is given by Equation (5).

(3) $$\overset{=}{\tau }=\mu \left[\left\{\nabla \vec{v}+\nabla {\vec{v}}^{T}\right\}-\frac{2}{3}\nabla \cdot \vec{v}I\right]$$

where,

*I*: unit tensor;
*μ*: molecular viscosity;
$\nabla \cdot \vec{v}I$: the result of volume dilation.

Further, Equation (6) represents the energy equation:

(4) $$\frac{\partial }{\partial t}\left(\rho E\right)+\nabla \cdot \left\{\vec{v}\left(\rho E+p\right)\right\}=\nabla \cdot \left\{k_{eff}\nabla T-\underset{j}{\sum }h_{j}{\vec{J}}_{j}+\left({\overset{=}{\tau }}_{eff}\cdot \vec{v}\right)\right\}+S_{h}$$

where,

${\vec{J}}_{j}$: diffusion flux of species *j*;
*S_h_* is a volumetric heat source term;
*k_eff_*: effective conductivity (*k + k_t_*, where *k_t_* is the turbulent thermal conductivity, defined according to the turbulence model being used).

The molten metal solidification was quantitatively determined using the enthalpy-porosity approach the mushy zone, or the liquid portion of molten metal in the range of 0 to 1, was regarded as a porous media. The liquid percentage from each element has been calculated considering its porosity. Additionally, the semi-molten elements were handled as if they had a porosity percentage of one and were non-porous. Therefore, it was thought that the velocities of fully cemented cells were zero. Additionally, a “pseudo” porous media was developed to represent the mushy zone. This indicates that the porosity percentage in the mushy zone ranged from 0 to 1 [38]. In this model, metal enthalpy has been calculated by adding the sensible enthalpy, h, and the latent gas, Δ*H*:

(5) H=h+ΔH

where

$$h=h_{ref}+\int_{T_{ref}}^{T}C_{p}dT$$

In addition, *h_ref_* stands for reference enthalpy, *T_ref_* for reference temperature, and *C_p_* for constant pressure specific heat. Additionally, the liquid fraction, £, is defined as

$$£=0\;if\;Temperature\;\left(To\right)<Solidus\;Temperature\left(Ts\right)$$

$$£=1\;if\;Temperature\;\left(To\right)>Solidus\;Temperature\left(Ts\right)$$

$$£=\frac{To-Ts}{Tl-Ts}\;if\;Ts<T<Tl$$

The latent heat content of the molten metal is represented as *L*, *H* = £. The latent heat content for both solids and liquids can range from 0 to 1. The value of latent heat value can be obtained from reference [39]. Additionally, the following is how the energy equation for solidification/melting problems is expressed:

(6) $$\frac{\partial }{\partial t}\left(\rho H\right)+\nabla \cdot \left(\rho \vec{v}H\right)=\nabla \cdot \left(k\nabla T\right)+S$$

where *S* is the source term and *H* is the enthalpy. The fundamental strategy used to handle melting and solidification is to modify the thermal energy equation by including a phenomenological heat source factor. The domain was initially initialized with a certain temperature of molten steel at the start of the calculation. The beginning circumstances are used to determine the heat source term. The local mass fraction of molten steel, sulfur material, and *Y_i_* is predicted by the solution of a convection–diffusion equation for the *ith* species. The conservation formula for all liquid phases is as follows:

(7) $$\frac{\partial }{\partial t}\left(\rho Y_{i}\right)+\nabla \cdot \left(\rho \vec{v}Y_{i}\right)=-\nabla \cdot {\vec{J}}_{j}$$

Therefore, one of the components of micro-alloyed steel is sulfur, and one goal of this work is to comprehend how alumina inclusions move when there is interfacial tension. Sulfur is dispersed in steel in this situation; hence, the diffusion coefficient may be calculated using the following equation [40]:

(8) $$D=\frac{kT}{2\pi \mu d}{\left[\frac{m_{1}+m_{2}}{2m_{2}}\right]}^{\frac{1}{2}}$$

where *d* is the diameter of atoms and *m*_1_ and *m*_2_ are the atomic mass of the solute and solvent, respectively. *T* is the temperature of the melt, *μ* is the viscosity of the molten metal, and *k* is Boltzmann’s constant (1.38 × 10^−23^ J/K).

For multiphase simulations with precisely defined immiscible incompressible fluid interfaces, the volume of fluid (VOF) model [54,55] was utilized. In this work, two immiscible fluids were used: molten steel and alumina inclusions. All processes share two variables, pressure and velocity, which are represented by volume-averaged values. To create a single set of equations, the mathematical equations were immediately volume-averaged. The fluid interface was then observed using the color function *Ψ*, which is described as follows. When *Ψ* = 1, it is presumed that just the first phase has filled the control volume. When *Ψ* = 0, however, it is presumed that the second phase has already filled the control volume. However, it is assumed that the element has an interface and divides into two distinct phases when the criterion of 0 < *Ψ* < 1 is satisfied. Additionally, a numerical method involves calculating the following transport equation to determine the fluid front:

(9) $$\frac{\partial F}{\partial t}+u\cdot \nabla F=0$$

Here, *F* is the volume fraction of the fluid in a cell and *u* is the flow velocity vector.

In this study, geo-reconstruct advection techniques were employed. It is generally known that the discretization of the governing equations has a significant influence on the device representation. Thus, the explicit scheme combined with the geo-reconstruct interface interpolation strategy produced the two-dimensional problem’s solution. The volume fraction values computed at the preceding time point in the explicit approach are added to the range of values covered by standard finite-difference interpolation techniques.

(10) $$\frac{\alpha_{q}^{n+1}\;\rho_{q}^{n+1}-\alpha_{q}^{n}\rho_{q}^{n+1}\;}{\Delta t}V+\sum_{f}\left(\rho_{q}U_{f}^{n}\alpha_{q\;,f}^{n}\right)==\left[\overset{n}{\underset{p=1}{\sum }}\left({\overset{\cdot }{m}}_{pq}-{\overset{\cdot }{m}}_{qp}\right)+S_{\alpha_{q}}\right]V$$

### 2.2. Computational Details

**Figure 4:**
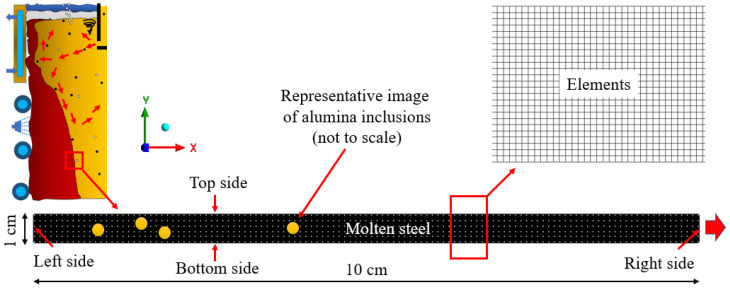
A small-scale domain considered for the study of motion of an inclusion.

A numerical model has been created in the current study to simulate melt flow and forecast inclusion motion characteristics in continuous casting molds. In this study, a tiny region within the continuous casting mold that is close to the solid–liquid boundary has been used for CFD simulation. Using Ansys Fluent, a transient two-dimensional, two-phase numerical model was created (Academic version: 21.0, ANSYS, Inc., Canonsburg, PA, USA). In this study, we ignored the aggregation phenomena of inclusion particles and assumed that inclusion particles have a spherical form. A schematic design of the domain, information about the phases, and a meshed zone with dimensions are shown in Figure 4. From Siddiqui et al. [26,50] work, the thermo-mechanical characteristics for the modeling were obtained. Convective boundaries with appropriate heat transfer coefficients were thought to exist at the domain’s perimeter. The grid-independence test has been reported in our previous work [50]. It was concluded that approximately 200,000 elements gave suitable results as compared to a higher number of elements. By taking an average temperature reading at the mid-vertical plane of the domain, the convergence of the solution was investigated. From [50], it was possible to determine the thermo-physical characteristics of molten steel and alumina inclusions. The equation was utilized in the simulation to account for the surface tension correlation between molten steel and alumina (23). A computational model was also used to measure the interfacial tension between molten steel and alumina inclusions using Equation (12). We took advantage of empirical relationships from the work of Jeong et al. [26]. The surface tension of SPFH590 steel and the interfacial tension of SPFH590 steel and alumina inclusion were established experimentally. The formulas below can be used to determine the surface tension of SPFH590 steel:

(11) $$\sigma_{L}=\left(1511+0.08277\;T\right)-\left(1041-0.5156\;T\right)\times \left\{\ln\left[1+exp\left(-3.583+19,846/T\right)\left(wt.\%\;S\right)\right]\right\}$$

where $\sigma_{L}$ is surface tension, *T* is temperature in K, and *S* is the sulfur concentration in ppm.

The following equation describes the interfacial stress between SPFH590 steel and an alumina inclusion.

(12) $$\sigma_{PL}=\left\{3050.51+131,437.97\times \left(wt.\%\;S\right)-1.544\times 10^{7}{\left(wt.\%\;S\right)}^{2}\;-3.378\times 10^{9}\;{\left(wt.\%\;S\right)}^{3}\right\}\;+\left\{-0.8498-79.739\times \left(wt.\%\;S\right)+7655.06\times {\left(wt.\%\;S\right)}^{2}\;+1.962\times 10^{6}\times {\left(wt.\%\;S\right)}^{3}\right\}T$$

where $\sigma_{PL}$ is interfacial tension between SPFH590 steel and an alumina inclusion.

A two-phase simulation of molten steel combined with sulfur and alumina was chosen to employ the volume of fluid (VOF) approach, and the surfactant concentration was created using a different species model (sulfur in this study). The interfacial tension that develops between molten metal and alumina inclusions varies depending on the temperature gradient and sulfur content. Due to the size of the domain and viscosity of liquid steel close to the border of solidification, the whole melt flow was considered to be a laminar flow for the sake of problem simplification (solid–liquid interface). Additionally, the heat transfer coefficient between molten metal and air was examined without taking into account any radiation from the molten metal. Additionally, the domain’s original temperature was kept constant during the simulation. We also looked at the motion of the inclusion particle close to the border of the solidifying molten steel. Surfactant concentration and temperature affect the Marangoni forces, or interfacial tension, on inclusion particles. To better comprehend the behavior of inclusion particles near the hardening metal borders, we used sulfur as a surfactant for our investigation. The area that was taken into consideration for the investigation of an inclusion particle’s transportation behavior during solidification is depicted in Figure 4. The top, bottom, and left sides are adiabatic and there is no heat or mass flow as shown in Figure 4. In addition to this, the right side wall is not insulated and heat dissipation occurred from this side. The representative inclusions have also been shown in the domain. The alteration in interfacial tension may cause the inclusion particles to be pushed and engulfed in solidifying borders.

In order to attain the sulfur content throughout the domain, the species model was also used. This numerical model also took the solidification concept into account. The species continuity equation was solved at each time step during the simulation’s two transient periods. The numerical model for a molten steel solidification has been addressed in our prior work [50] and confirmed using the numerical findings of Cho et al. [56]. The profile of the shell’s thickness was compared to the findings of expected computer calculations. The anticipated outcomes appear to be in strong agreement with the work of Cho et al. [56].

## 3. Results and Discussion

### Effect of Interfacial Tension

**Figure 5:**
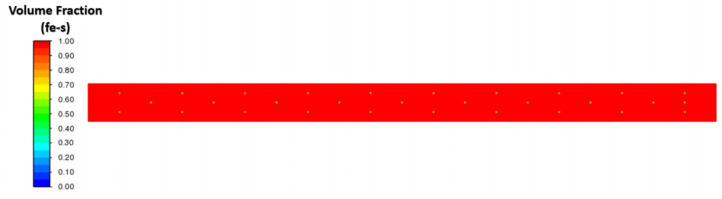
Two-phase model of inclusion and molten steel shown at the beginning of simulation.

**Figure 6:**
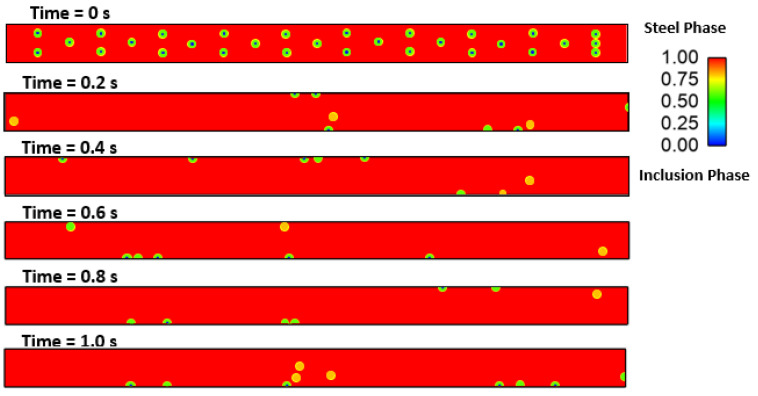
Motion of alumina inclusion during solidification (initial temp: 1893 K, sulfur: 10 PPM).

**Figure 7:**
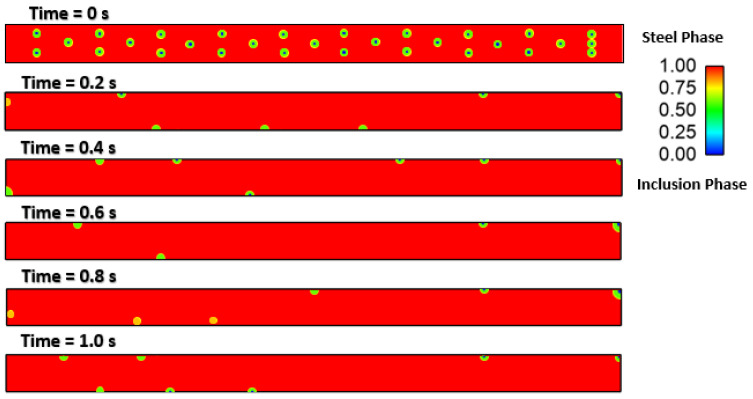
4.29: Motion of alumina inclusion during solidification (initial temp: 1893 K, sulfur: 40 PPM).

**Figure 8:**
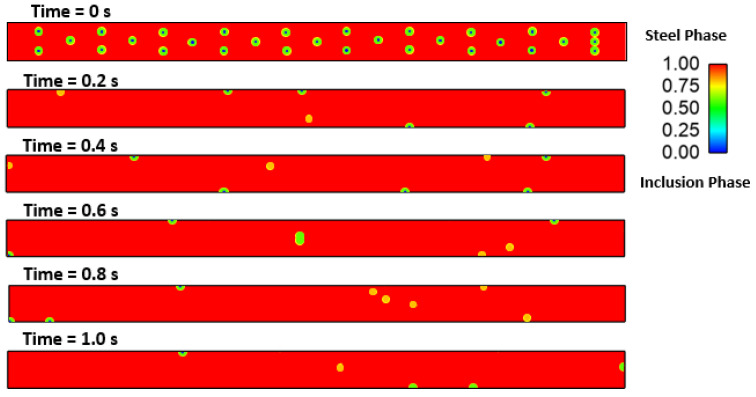
4.30: Motion of alumina inclusion during solidification (initial temp: 1893 K, sulfur: 70 PPM).

**Figure 9:**
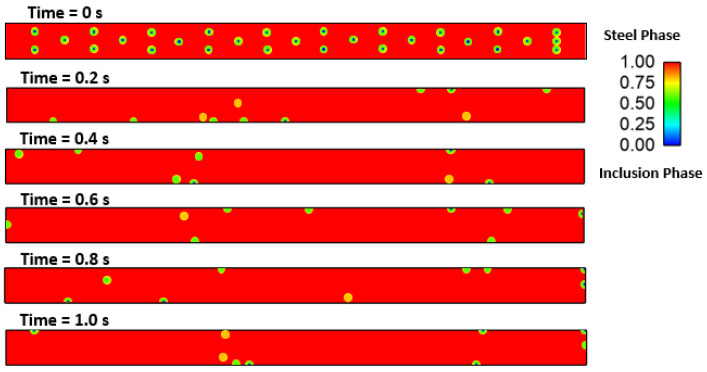
4.31: Motion of alumina inclusion during solidification (initial temp: 1893 K, sulfur: 100 PPM).

We investigated the effect of interfacial tensions on alumina inclusions. We have previously mentioned [50] the effect of sulfur concentration on interfacial tension rises at the solidification boundary (near solid–liquid interface). The empirical relationship mentioned in Equations (11) and (12) has been used in this simulation. A proper heat transfer condition was given to boundaries as shown in Figure 4. We have observed that the inclusions are more concentrated towards the solidifying shell in the continuous casting mold. To know more details about the inclusion motions near the solid–liquid boundaries, we have studied a small zone where a much larger temperature and concentration gradient of sulfur is present. Figure 5 shows a small domain discretized in 60,000 elements and having dimensions of length: 10 cm and width: 1 cm. We carried out a 2-dimensional, two-phase simulation namely, (a) molten steel mixed with sulfur and (b) an inclusion. We have used four different cases of sulfur concentration in this simulation namely 10, 40, 70, and 100 PPM. The initial temperature of the molten zone was 1893 K and inclusions having diameter of 300 µm were placed in certain places (as shown in Figure 5) at the beginning of the simulation. The inclusions were considered as spherical/circular in shape. The domain velocity was zero at the start of simulation. The properties of steel and alumina were kept similar to the first part of the research work. As the simulation started, we observed alumina inclusion movement in the domain due to the change in interfacial tension and temperature (Marangoni force) in the domain. It is evident from Figure 6, Figure 7, Figure 8 and Figure 9 that the inclusions have been moved to other locations when the temperature gradient and interfacial tension gradient existed. Secondly, the inclusion particles were concentrated towards the boundary where the temperature gradient was higher. In addition, it is an evident from Figure 6 and Figure 9 that an increase in sulfur content from 10 ppm to 40 ppm resulted in more rapid dislocations/movements of the alumina inclusions. During initialization of the solution, we defined the positions of inclusions at certain and specific locations. Once the solver started solving the heat and mass transfer equations along with solidification conditions, we observed formation of interfacial gradients due to the change in temperature in the domain. In addition to this, sulfur concentration also affected the interfacial tension gradient. It was also observed that the motions of inclusions are rapid and random. The above-mentioned phenomenon suggests that an increased concentration of sulfur (from 10 to 70 ppm) has resulted in higher interfacial tension and caused faster engulfment of sulfur. Nevertheless, inclusion motion seemed less affected when sulfur concentration was 100 ppm. Thus, it can be said that highest number of inclusion can be trapped at the solidifying boundary when 70 ppm sulfur is present in the molten steel. However, any further increase in sulfur concentration has opposite effect as was also depicted in the experimental work of Jeong et al. [26]. We observed many inclusions particles close and nearby to each other. Therefore, during enlargement, we cannot show all inclusions at same time. Secondly, the motions of the alumina will be chaotic at any instant because of applied forces. The motions are in both directions (x and y). Thus, it is possible that inclusions are overlapping at any instant of time. Sometimes, it moved in the x-direction. The current images show how the concentration of sulfur can produce interfacial tension gradients which affects the motions of inclusions in liquid steel.

**Figure 10:**
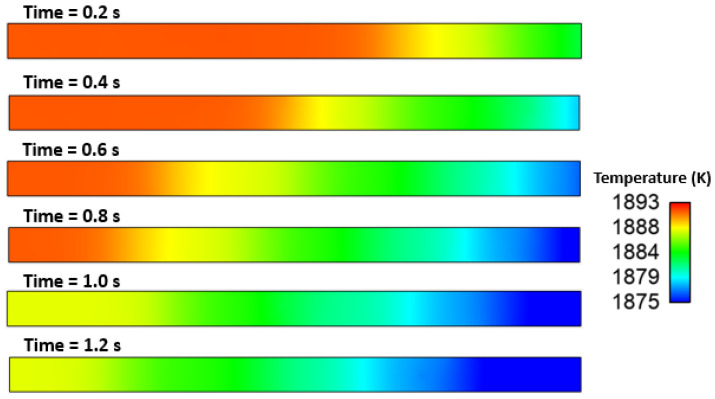
Temperature distribution in the domain (S: 10 PPM).

**Figure 11:**
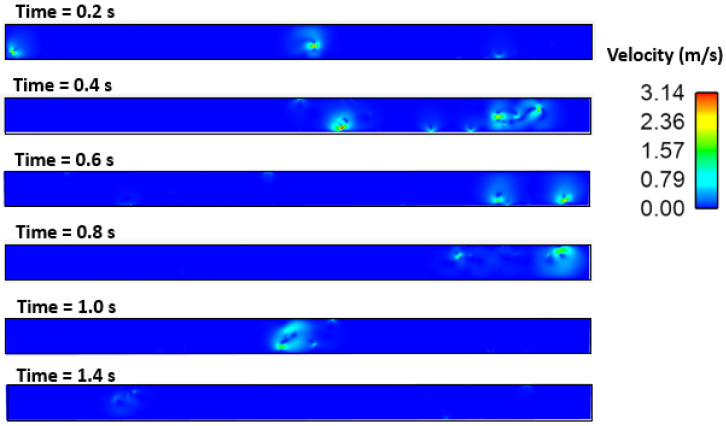
Velocity field of inclusion and molten steel (S: 10 PPM).

**Figure 12:**
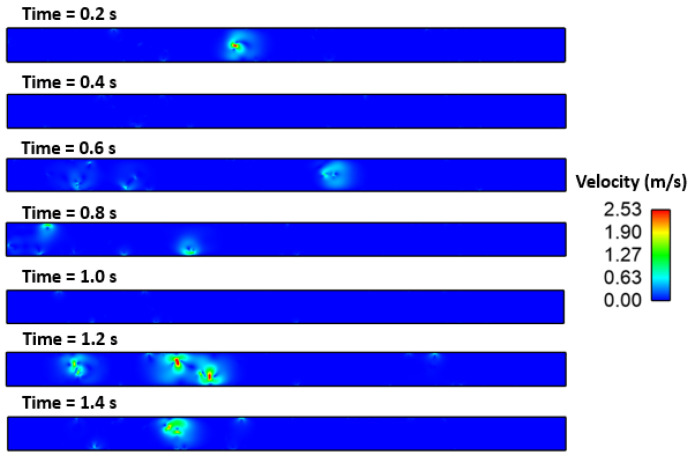
Velocity field of inclusion and molten steel (S: 40 PPM).

**Figure 13:**
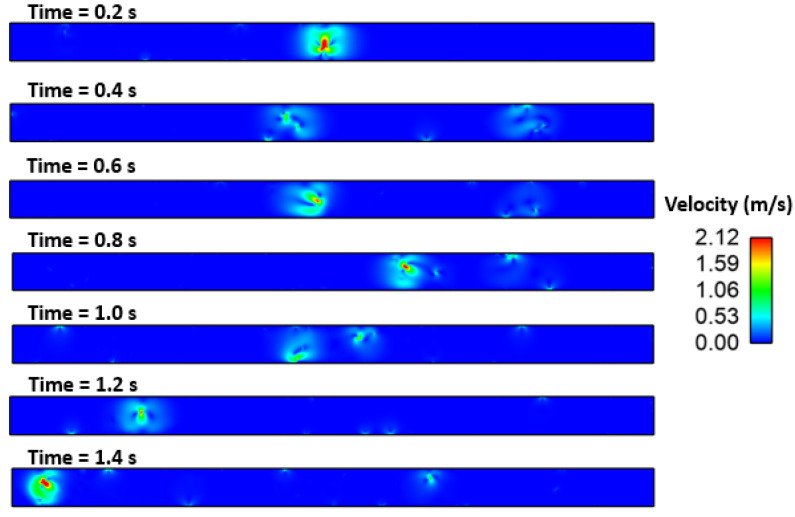
Velocity field of inclusion and molten steel (S: 70 PPM).

**Figure 14:**
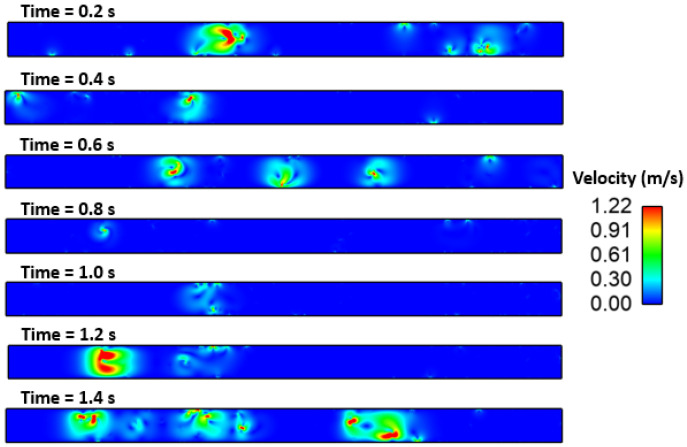
Velocity field of inclusion and molten steel (S: 100 PPM).

Figure 10, Figure 11, Figure 12, Figure 13 and Figure 14 show the velocity filed generation due to different sulfur content. It is interesting to see the velocity filed was induced due to the Marangoni force. In this simulation domain, melt flow takes place due to the viscosity of the molten steel at a particular instant and location. Further, interfacial tension gradient was caused not only by differences in temperature, but also by the concentration of sulfur in the domain. It can be noted that the velocity field was generated near inclusions where interfacial tension is induced due to the temperature and sulfur gradient. It can be noted that the velocity of melt and inclusion in the domain is varied by sulfur concentration. Further, the domain which has less sulfur has a higher velocity magnitude as compared to other domains. Secondly, the heat transfer boundary conditions were the same for all four cases. Hence, the possible cause of this variation in flow can be attributed to the interfacial tension. Moreover, the velocity profile suggests and affirms that the motion of inclusion particles is dependent on the surfactant (sulfur) concentration gradients. Hence, it can be concluded that inclusions are prone to move towards the solidified shell when the surfactant (sulfur) concentration is higher.

**Figure 15:**
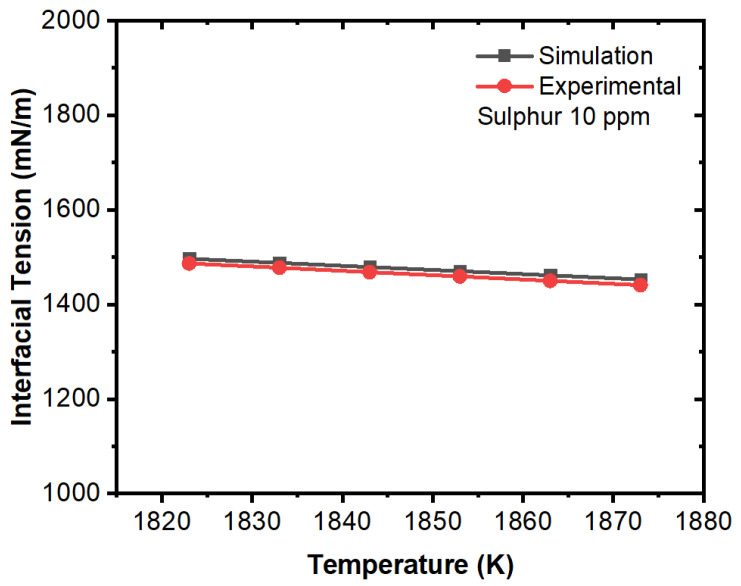
Comparison of experimental and simulation results of interfacial tension with respect to temperature (sulfur: 10 ppm, experimental work by Jeong et al. [26]).

**Figure 16:**
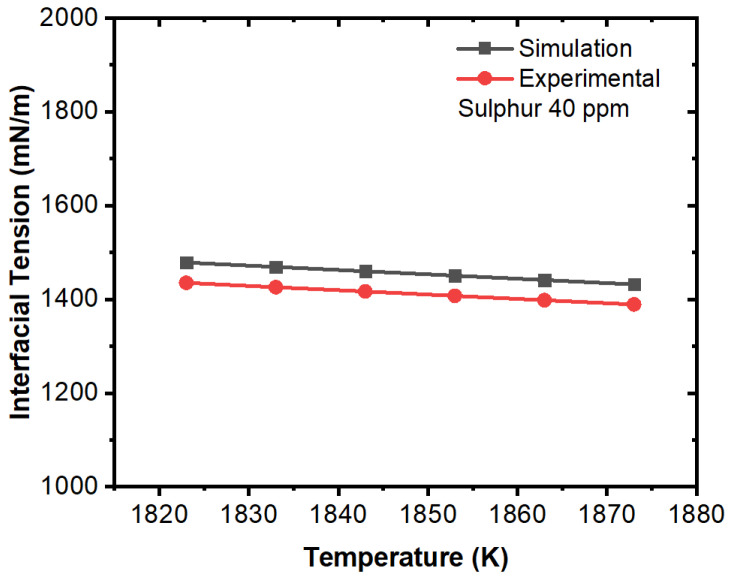
Comparison of experimental and simulation results of interfacial tension with respect to temperature (sulfur: 40 ppm, experimental work by Jeong et al. [26]).

**Figure 17:**
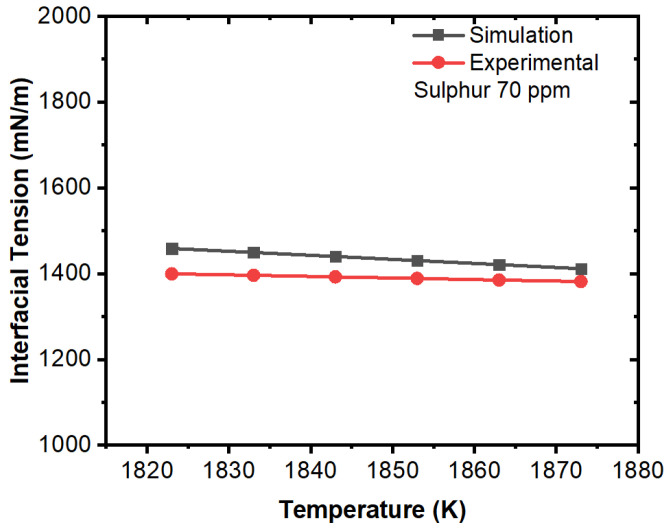
Comparison of experimental and simulation results of interfacial tension with respect to temperature (sulfur: 70 ppm, experimental work by Jeong et al. [26]).

**Figure 18:**
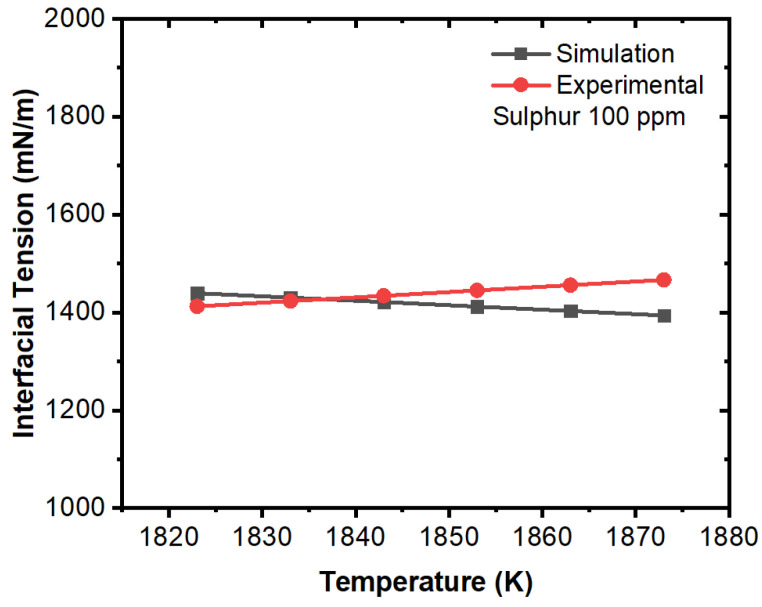
Comparison of experimental and simulation results of interfacial tension with respect to temperature (sulfur: 100 ppm, experimental work by Jeong et al. [26]).

**Figure 19:**
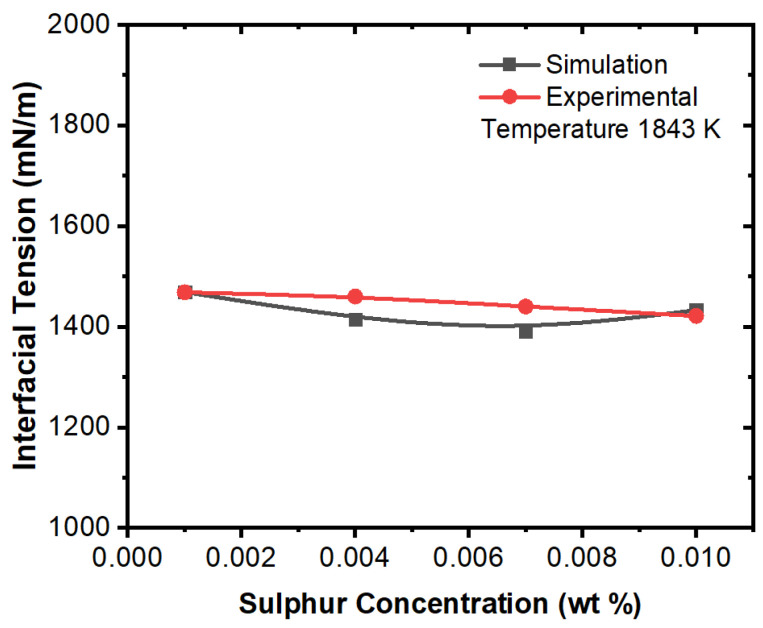
Comparison of experimental and simulation results of interfacial tension with respect to sulfur concentration (temperature 1843 K, experimental work by Jeong et al. [26]).

**Figure 20:**
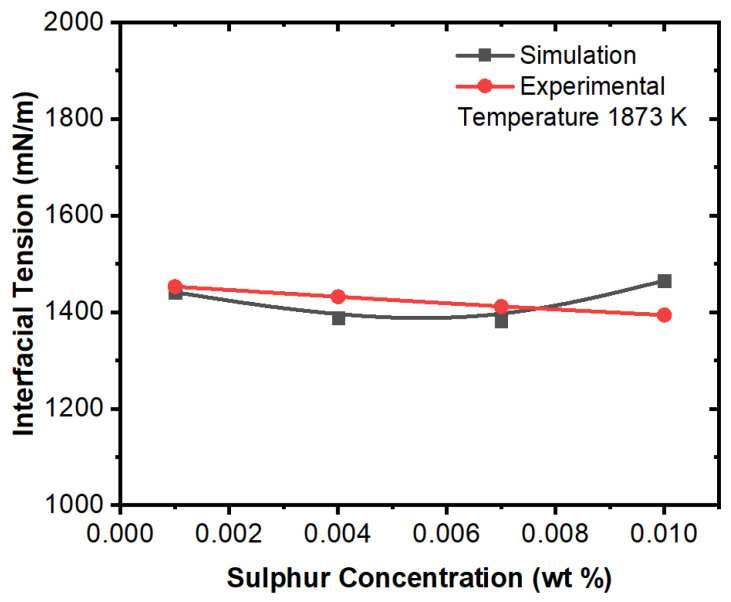
Comparison of experimental and simulation results of interfacial tension with respect to sulfur concentration (temperature 1873 K, experimental work by Jeong et al. [26]).

Further, we compared the experimental and simulation results for interfacial tension in the domain (solid–liquid boundary) at different concentrations of sulfur. The comparative results are illustrated in Figure 15, Figure 16, Figure 17, Figure 18, Figure 19 and Figure 20. The experimental values have been calculated from the correlation developed by Jeong et al. [26] which has been mentioned in Equations (11) and (12). For the numerical simulation, we utilized the data from the numerical solution and then we calculated interfacial tensions. It can be noted that the simulation values were quite similar to the experimental results. A certain difference in values was observed in the 100 ppm case; however, this can be justified due to the characteristic change in interfacial tension after 70 ppm as per the experimental results. The other important fact is that the interfacial tension near the solid–liquid boundary increased with respect to decreases in temperature up to 70 ppm except for the 100 ppm case. Thus, it can be said that temperature plays an important role on the motion of inclusions and the solidification process induces higher Marangoni forces/interfacial tension that results in a larger percentage of inclusion entrapment in the solidifying shell.

Figure 19 and Figure 20 show effect of sulfur concentration on interfacial tension at certain temperatures. It can be noted here that an increase in sulfur concentration has opposite impact on interfacial tension between alumina inclusions and molten steel. This also evident from the experimental work of Jeong et al. [26]. Further, it was also reported that sulfur may preferentially concentrate near the interface during solidification, resulting in a concentration differential in the boundary layer. This phenomenon is also evident from the numerical results that a surfactant concentration gradient existed near the boundary layer and a higher amount of inclusion particles moved towards the solidifying boundary. Lastly, it can be correlated here that in this simulation, the alumina inclusions encounter lower interfacial tension from the solidifying boundary. Thus, it can be entrapped under these conditions. Finally, in practical situations, dendrites were formed at the solid–liquid boundary, and thus alumina inclusions may be caught between the dendrite arms.

## 4. Conclusions

A numerical model was developed to study alumina inclusion motion behavior in continuous casting molds. We investigated inclusion motion under the influence of interfacial tension in small domains of solidifying steel. For this purpose, we used a two-phase VOF model for the simulation. The effect on motion due to surfactant concentration (sulfur) on alumina inclusion has been studied. The first phase considered a mixture of molten steel and sulfur and the second phase considered alumina inclusions. The empirical formula for interfacial tension was applied in the computation. The following are the main conclusions drawn from the present investigation:

- The Marangoni effect in the mold region was induced by the difference in temperature and sulfur content. The projected results demonstrate that inclusions were susceptible to engulfment by the solidification front under the influence of a larger surface tension differential between the inclusions and melt.
- Interfacial tension had a considerable impact on the motion behavior of the inclusion particle. It was observed that the inclusion movement at the solid–liquid interface was influenced by the sulfur content of the micro-alloyed steel. It was also observed that the moving solid contact captured inclusion particles.
- The projected results demonstrated that inclusions were susceptible to being engulfed by the solidification front under the influence of a larger surface tension differential between the inclusions and melt. A rise in sulfur content (to 100 ppm) had no discernible effect.
- We concluded that sulfur may preferentially concentrate near the interface during solidification, resulting in a concentration differential in the boundary layer. This phenomenon showed agreement with the numerical results, where a surfactant concentration gradient existed near the boundary layer and a higher amount of inclusion particles moved towards the solidifying boundary.

## Figures and Tables

**Table 1:** Automotive steel (SPFH590) chemical composition (in weight%).

Element	C	Mn	Si	P	Al	Nb
Concentration	≤0.1	≤3.0	≤0.5	≤0.1	≤0.1	≤0.1

**Table 2:** Thermophysical properties of SPFH590 steel.

Parameters	Values
Density of molten steel [51]	ρ (kg m^−3^) = 8621.17 − 0.88 T
The viscosity of molten steel [51]	μ (mPa s) = 0.2388*exp^(47.44/(RT))^
Specific heat [52]	750 J kg^−1^ K^−1^
Thermal conductivity [52]	41 W m^−1^ K^−1^
Surface tension (*σ_L_*) and interfacial tension (σ_PL_)	Shown in Equations (11) and (12) [25,26]
Solidus temperature	1781 K
Liquidus temperature	1798 K
Alumina inclusion size	300 μm

* where μ is the viscosity, R is the molar gas constant, and T is the absolute temperature (K).

**Table 3:** Process parameters [28,50,52].

Process Parameters	Values
Mold width	1500 mm
Mold length	3600 mm
SEN submergence depth	160 mm
Nozzle port downward angle	15 degree
Inlet velocity	2 m/s
Outlet	Pressure outlet condition
Alumina inclusion density	2500 kg/m^3^
Shell surface temperature	1273 K
Mold conductivity	315 W/mk
Latent heat	272,000 (J/kg)

## Data Availability

Available on request.
